# Identification of small molecule compounds that inhibit the HIF-1 signaling pathway

**DOI:** 10.1186/1476-4598-8-117

**Published:** 2009-12-09

**Authors:** Menghang Xia, Kun Bi, Ruili Huang, Ming-Hsuang Cho, Srilatha Sakamuru, Susanne C Miller, Hua Li, Yi Sun, John Printen, Christopher P Austin, James Inglese

**Affiliations:** 1 NIH Chemical Genomics Center, National Institutes of Health, Bethesda, MD 20892-3370, USA; 2 Invitrogen - Discovery Sciences, 501 Charmany Dr., Madison, WI 53719, USA; 3 Department of Radiation Oncology, University of Michigan, Comprehensive Cancer Center, Ann Arbor, MI, USA

## Abstract

**Background:**

Hypoxia-inducible factor-1 (HIF-1) is the major hypoxia-regulated transcription factor that regulates cellular responses to low oxygen environments. HIF-1 is composed of two subunits: hypoxia-inducible HIF-1α and constitutively-expressed HIF-1β. During hypoxic conditions, HIF-1α heterodimerizes with HIF-1β and translocates to the nucleus where the HIF-1 complex binds to the hypoxia-response element (HRE) and activates expression of target genes implicated in cell growth and survival. HIF-1α protein expression is elevated in many solid tumors, including those of the cervix and brain, where cells that are the greatest distance from blood vessels, and therefore the most hypoxic, express the highest levels of HIF-1α. Therapeutic blockade of the HIF-1 signaling pathway in cancer cells therefore provides an attractive strategy for development of anticancer drugs. To identify small molecule inhibitors of the HIF-1 pathway, we have developed a cell-based reporter gene assay and screened a large compound library by using a quantitative high-throughput screening (qHTS) approach.

**Results:**

The assay is based upon a β-lactamase reporter under the control of a HRE. We have screened approximate 73,000 compounds by qHTS, with each compound tested over a range of seven to fifteen concentrations. After qHTS we have rapidly identified three novel structural series of HIF-1 pathway Inhibitors. Selected compounds in these series were also confirmed as inhibitors in a HRE β-lactamase reporter gene assay induced by low oxygen and in a VEGF secretion assay. Three of the four selected compounds tested showed significant inhibition of hypoxia-induced HIF-1α accumulation by western blot analysis.

**Conclusion:**

The use of β-lactamase reporter gene assays, in combination with qHTS, enabled the rapid identification and prioritization of inhibitors specific to the hypoxia induced signaling pathway.

## Background

The maintenance of oxygen homeostasis is essential for the human body. Hypoxia, defined as a reduction in the normal level of tissue oxygen tension, is associated with cancer, inflammation and ischemia [1]. The transcriptional factor hypoxia-inducible factor 1 (HIF-1) is critical in responding to hypoxic environments by inducing survival and anti-apoptotic genes. HIF-1 is composed of two subunits: hypoxia-responsive HIF-1α and constitutively-expressed HIF-1β (also known as ARNT, aryl hydrocarbon receptor nuclear translocator) [2]. Under normal oxygen tension, HIF-1α is rapidly degraded by the ubiquitin-proteasome pathway [3,4], but under hypoxic conditions, HIF-1α is stabilized by the attenuation of prolyl hydroxylase activity [5,6]. The accumulated HIF-1α heterodimerizes with HIF-1β and translocates into the nucleus. The HIF-1 complex binds to a hypoxia-response element (HRE), composed of a core 5'-ACGTG-3' sequence, in concert with the transcriptional coactivator p300/CBP [7], thereby activating the expression of target genes, such as vascular endothelial growth factor (VEGF) [8], erythropoietin [9], and the glucose transporters GLUT1 and GLUT3 [10,11].

In many solid tumors, intratumor hypoxia up-regulates HIF-1α expression, a response that is correlated with increased angiogenesis, oncogenesis, and poor cancer prognosis [12]. In HIF-1α knockout mice, loss of HIF-1α in embryonic stem cells and endothelial cells dramatically retards solid tumor and blood vessel growth, and a reduced capacity to release angiogenic VEGF during hypoxia [13,14]. Therefore, HIF-1 responsive tumor hypoxia has become the focus of active biomedical investigations and its inhibition is emerging as a potentially valuable and novel approach to cancer therapy. Several small molecule inhibitors of HIF-1α activity are entering clinical development [15-17], such as 2ME2 (2-methoxyestradiol), an inhibitor of microtubule polymerization, 17-AAG (17-allylamino-17-demethoxygeldanamycin), a HSP90 inhibitor, topotecan, a topoisomerase I inhibitor, and PX-478 (S-2 amino -3- [4'-N,N,-bis (2-chloroethyl) amino] phenyl propionic acid N-oxide dihydrochloride). These compounds were reported to either inhibit intracellular HIF-1α level or induce HIF-1α degradation [17]. Several compounds have been in clinical trials, but none appear very promising due to lack of target specificity and low clinical efficacy [17].

In order to rapidly identify potent and specific inhibitors of the HIF-1 pathway we developed a cell-based HIF-1 mediated β-lactamase reporter gene assay and used a quantitative high-throughput screening (qHTS) [18,19] approach to test 73,000 compounds. The compounds identified from the screen were further confirmed in several follow-up studies including VEGF secretion assay. This approach allowed us to rapidly and efficiently identify small molecule inhibitors of the HIF-1 signaling pathway.

## Results

### Development and validation of a hypoxia responsive β-lactamase reporter gene assay

**Figure 1:**
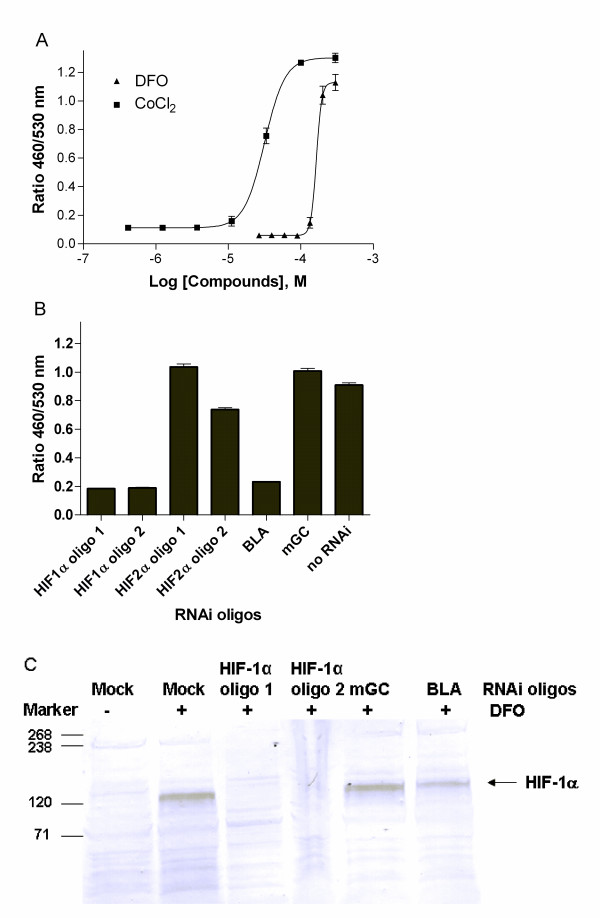
**A. HRE-*bla *ME-180 cells express β-lactamase in response to hypoxia**. Cells were incubated with indicated amount of DFO or CoCl_2 _for 17 hrs before β-lactamase assay was performed. **B. HIF1α, but not HIF2 α, mediates the expression of β-lactamase in response to hypoxia**. Cells were incubated with indicated Stealth RNAi™ duplexes for 55 hrs, and then treated with 125 μM CoCl_2 _for 17 hrs before β-lactamase assay was performed. **C. Western blot analysis for DFO-induced HIFα accumulation and RNAi knockdown**. Cells were incubated with indicated Stealth RNAi™ duplexes for 55 hrs, and then treated with 250 μM DFO for 5 hrs before HIF1α western blot was performed as described in Materials and Methods.

We have generated a HRE-*bla *line in ME 180 cells, human cervical cancer cells, by isolating a clonal cell line that responds to hypoxic conditions by up-regulating β-lactamase expression after fluorescence-activated cell sorting (FACS) of cells in response to treatment with deferoxamine (DFO), a known inducer of HIF-1 [20]. This line expressed β-lactamase in response to DFO and cobalt chloride (CoCl_2_) (Figure 1A), both of which displace iron from the prolyl hydroxylases F1H1 and PHD1-3, causing its inhibition, to mimic the induction of hypoxic conditions [21], with EC_50s _of 164 μM for DFO and 32 μM for CoCl_2_, respectively. This response was also time dependent with maximal β-lactamase expression after cells were treated with DFO for 17 hrs (data not shown). The HRE β-lactamase reporter gene assay (HRE-*bla *assay) was further miniaturized into 1536-well plate format. In this format, CoCl_2 _dose-dependently induced β-lactamase expression with an EC_50 _of 54 μM, similar to that measured in 384-well plate.

To date, three HIFα isoforms have been described, with the best characterized being HIF-1α and HIF-2α. In cancer, the HIF system is up regulated both by microenvironmental hypoxia and by genetic events that lead to enhanced translation or stability of HIF-1α[17]. While HIF-1α has been shown to mediate hypoxia-induced responses in ME-180 cells [22], we validated the HRE-*bla *assay using HIF-1α and HIF-2α specific RNAi and examined the effect on HRE-*bla *activity. Incubation of cells with HIF-1α RNA duplexes blocked hypoxia-induced β-lactamase expression, whereas HIF-2α duplexes had little effect (Figure 1B and 1C). This result suggests that HIF-1α, but not HIF-2α, regulates HIF-mediated gene expression in ME-180 cells.

### Identification of small molecule inhibitors of HIF-1 pathway using qHTS

**Figure 2:**
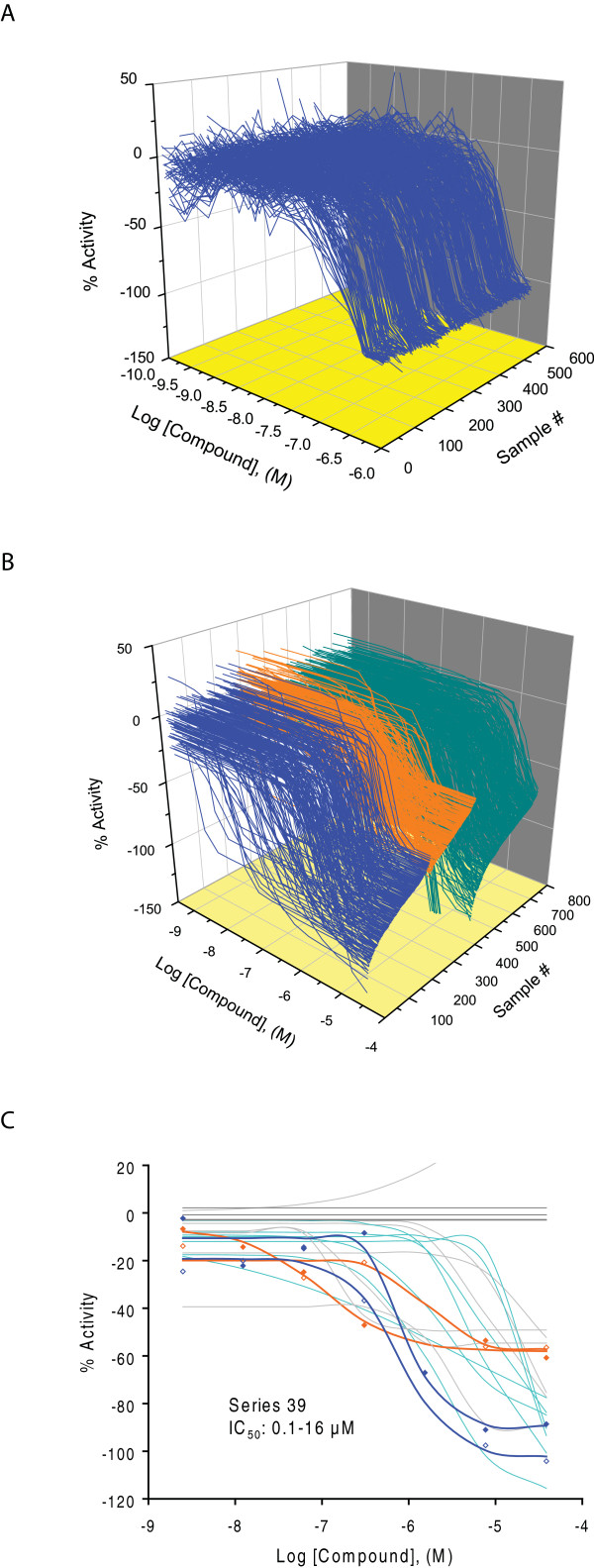
**A. Intra-plate concentration response titration curves for NSC 607097 in the screening**. Each curve was measured in duplicate in column 1 of each assay plate. B. qHTS concentration response data binned into curve classes 1-3 (Class 1 curves, blue; Class 2 curves, orange; Class 3 curves, green). C. Concentration response curves for compounds in series 39 (Class 1.1 curves, blue; Class 1.2 curves, orange; Class 2.1 curves, green; Class 3 curves, light gray; Class 4 curves, dark gray).

In the primary qHTS, 73,000 compounds were screened at 7-15 concentrations ranging from 0.5 nM to 38 μM in the HRE-*bla *assay. The inhibitory effect of the compounds was measured in the presence of 60 μM of CoCl_2 _in the screen. NSC 607097, a known inhibitor of HIF-1 pathway [23], was used as a positive control to examine the quality of the results from each plate. The NSC 607097 concentration response curves reproduced well in all 500 plates with an average IC_50 _of 0.29 ± 0.8 μM (Figure 2A). The average signal to background ratio was 4.1 and Z' factor averaged 0.7 for the entire screening. These data demonstrate that the qHTS platform is robust and suitable for identifying active compounds. Concentration-response data for all the compounds screened binned into curve classes are shown in Figure 2B.

In this primary qHTS (PubChem AID: 915), 350 inhibitors (0.5% of library) with class 1.1, 1.2, and 2.1 curves in the ratio readout (class 1-3 in the 460 nm reading and class 4 in the 530 nm reading) were identified. The distributions of curve class and potency for these compounds were listed in Table 1. A structure-activity relationship (SAR) analysis of these 350 compounds yielded 18 structural series each sharing a common scaffold (see Methods section for details). Ten of these 18 series and 6 singletons were selected for confirmation and follow up studies based on compound potency/efficacy ranges, curve quality and cytotoxicity. The concentration response curves for compounds in series 39 are showed in Figure 2C from the primary screen. Details on the procedure used for series prioritization can be found in the Materials and Methods section.

**Table 1:** Potency (half maximal inhibition concentration) distribution of active compounds in qHTS screening

	Number of active compounds in curve classes		
IC50 (μM)	1.1	1.2	2.1
< 0.10	4	12	0
0.10-1.0	16	33	7
1.0-10.0	60	40	86
10.0-100.0	2	2	88
Total	350		

**Figure 3:**
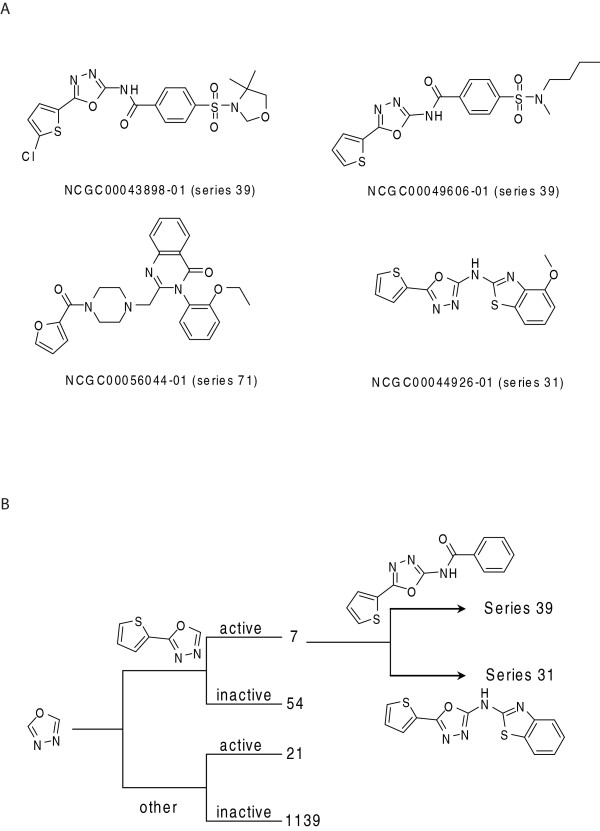
**Chemical structures of inhibitors for HIF-1 signaling pathway**. A. Four representative inhibitors from the qHTS. NCGC00044926 is a representative from series 31, NCGC00043898 and NCGC00049606 from series 39 and NCGC00056044 from series 71. B. Number of active and inactive compounds containing the 5-thiophen-1,2,4-oxadiazole core versus the 1,2,4-oxadiazole core. The thiophen-oxadiazole core appears to be important for compound activity. *Right*, Leadscope Maximal Common Substructures (MCS) used to populate series 31 and 39.

Among these we further identified compounds from three novel structural series (Figure 3A) that were the most potent and had the highest quality concentration-response curves for secondary testing in additional assays. The thiophen-oxadiazole core, rather than the oxadiazole group alone, seems to be important for compound activity in series 39 and 31. As shown in Figure 3B, of all the 64 compounds containing the thiophen-oxadiazole core, 7 (11%) were active and 54 were inactive; whereas of the 1329 compounds in the library containing only the oxadiazole moiety, only 21 (1.6%) were active. NCGC00044926 was assigned to a different series (series 31 rather than 39), even though it contains the thiophen-oxadiazole core as well. This is because NCGC00044926 also contains another scaffold, the methoxybenzothiazol-amino group, which is the common scaffold for series 31.

### Inhibition of CoCl2 and low oxygen induced HIF-1 signaling

**Figure 4:**
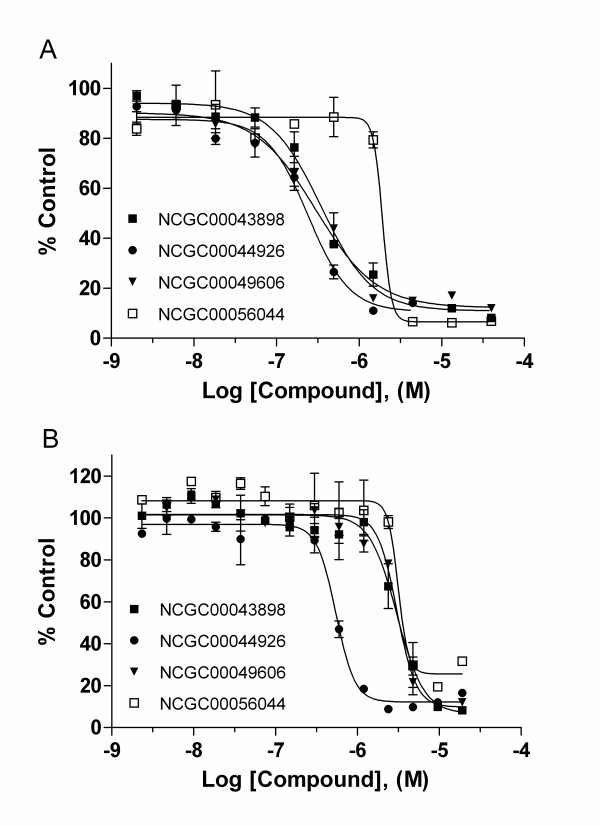
**Effect of four novel inhibitors on HIF-1 signaling pathway**. Inhibition of HIF-1 mediated β-lactamase activity in HRE-bla ME-180 cells by various concentrations of NCGC00043898, NCGC00044926, NCGC00049606 and NCGC00056044 in the presence of 1% oxygen (A) and 60 μM CoCl_2 _(B). The data are displayed as the percentage of control. Representative values are from a single experiment performed in duplicate. Data are expressed as mean ± SD.

In the confirmation study 40 compounds (Table S1, Additional file 1) from 10 series and 6 singletons were purchased and retested in the HRE-*bla *assay induced by CoCl_2_. Of the 40 compounds that had been tested in the primary screen, 36 showed the same activity in the confirmation assay giving a confirmation rate of 90%. To confirm that the compounds were specifically active against the HIF-1 mediated signaling pathway, we selected 10 from these 36 confirmed compounds based on potency and structure novelty. These compounds were tested in the same HRE-*bla *assay induced by low oxygen (≤1%) in a hypoxia chamber. All the compounds inhibited low oxygen-induced HIF-1 mediated signaling except for NCGC00078922. Compounds from series 31 were the most potent in both low oxygen and CoCl_2_-induced HRE-*bla *assays (Table 2). As shown in Figure 4A, NCGC00043898 (series 39), NCGC00044926 (series 31), NCGC00049606 (series 39) and NCGC00056044 (series 71) concentration-dependently inhibited HIF-1 mediated signaling induced by low oxygen. These compounds showed similar activities in a CoCl_2_-induced HRE-*bla *assay (Figure 4B). To study the mechanism involved in the inhibition of HIF-1 signaling, NCGC00043898, NCGC00044926, NCGC00049606 and NCGC00056044 were further examined for their effect on HIF-1α accumulation by western blot analysis. As shown in Figure S1 (Additional file 2), NCGC00043898 (series 39), NCGC00049606 (series 39) and NCGC00056044 (series 71) significantly inhibited CoCl_2 _induced HIF-1α accumulation at 10 μM and had even more inhibitory effect at 20 μM, whereas NCGC00044926 (series 31) showed little inhibition, which indicates this compound does not inhibit HIF-1α transcriptional activity via the inhibition of HIF-1α accumulation.

**Table 2:** Summary of compound activities (IC_50_, μM) in HRE-*bla *assay induced by ≤1% oxygen or CoCl2

Cluster	Compound ID	CoCl_2_	≤1% Oxygen
Singleton	NCGC00033933	0.013	0.84
Singleton	NCGC00053249	5.62	14.8
71	NCGC00056044	3.16	1.56
23	NCGC00078922	6.31	Inactive
31	NCGC00044926	0.50	0.17
31	NCGC00043631	0.50	0.11
27	NCGC00043836	2.51	0.39
39	NCGC00043898	2.82	1.22
39	NCGC00044763	2.51	1.10
39	NCGC00049606	3.16	0.75

In addition, the cytotoxicity of these compounds was investigated in a cell viability assay which measures intracellular ATP content. Only NCGC00056044 significantly decreased intracellular ATP content with an IC_50 _of 15.8 μM after a 17 hr treatment. None of the other compounds showed significant cytotoxicity. To further investigate the effect of these four compounds on the growth of ME 180 cells under normoxic or hypoxic (induction by CoCl_2_) conditions, intracellular ATP content was measured after compound treatment for 24 to 72 hr. After a 24 hr treatment, NCGC00056044 showed significant growth inhibition, while the others only showed minimal or no inhibitory effect (Table S2, Additional file 3). After 48-72 hr treatment all the compounds had an inhibitory effect on the growth of ME 180 cells. There was no significant difference of compound IC_50 _values with or without CoCl_2 _treatment.

### Inhibition of hypoxia induced VEGF secretion

**Figure 5:**
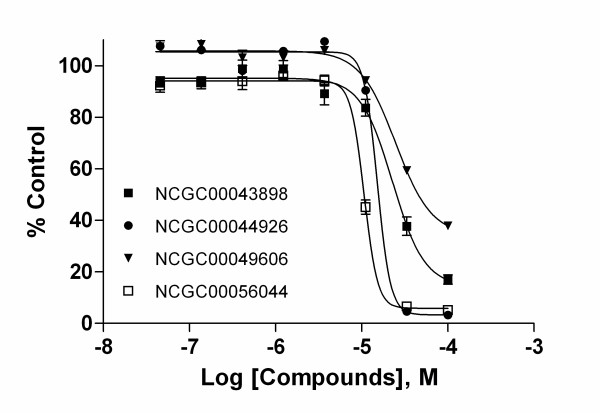
**Inhibition of hypoxia-induced VEGF secretion**. ME-180 cells were pre-treated with NCGC00043898, NCGC00044926, NCGC00049606 and NCGC00056044 at various concentrations for 5 min and then exposed to 15 μM iodochorohydrozyquinoline for 17 hrs. Then VEGF level was determined by human VEGF ELISA kit. Representative values are from a single experiment performed in duplicate. Data are expressed as mean ± SD.

To determine whether these compounds were modulating HIF-1 signaling, we evaluated the effect of theses compounds on VEGF secretion in ME180 cells. Expression of VEGF is induced in cells exposed to hypoxia [8], and VEGF is one of several well studied HIF-1 target genes [8,24]. Two known hypoxia inducers were used to induce VEGF secretion in the assays, CoCl_2 _and iodochorohydrozyquinoline [25]. As shown in Figure 5, NCGC00043898 (series 39), NCGC00044926 (series 31), NCGC00049606 (series 39) and NCGC00056044 (series 71) concentration-dependently inhibited iodochorohydrozyquinoline induced VEGF secretion, with IC_50_s of 23 μM, 15 μM, 25 μM and 10 μM, respectively. These compounds had a similar inhibitory effect on CoCl_2_-induced VEGF secretion (data not shown). However, a few compounds, including NCGC00033933 and NCGC00078922, had no effect on hypoxia induced VEGF secretion even though they showed inhibitory effects in both low oxygen and CoCl_2_-induced HRE-*bla *assays, suggesting that these compounds may affect HIF-1 mediated target genes other than VEGF, a result which warrants further investigation.

### Selectivity of compounds in HRE signaling pathway

Some of the compounds screened against the HIF-1 pathway were also tested in other signaling pathway assays at the NCGC. We compared the activity, in terms of potency and efficacy, of some of our confirmed active compounds including NCGC00043898, NCGC00044926, NCGC00049606 and NCGC00056044 in the HIF-1 pathway with their activities in other pathways including AP-1 and NFκB signaling. Among these four compounds, NCGC00044926 was inactive in both the AP-1-*bla *(PubChem AID: 357) and NFκB-*bla *assays. In the AP-1 pathway screen using a β-lactamase assay, NCGC00049606 and NCGC00056044 (PubChem AID: 357) showed partial efficacy with IC_50_s of 10 μM and 12.5 μM, respectively. The maximum inhibitory efficacies of NCGC00049606 and NCGC00056044 in the AP-1 pathway were 45% and 35%, respectively, whereas the inhibitory efficacies of these two compounds in the HIF-1 pathway were 91% and 80%. In the NFκB reporter gene assay, NCGC00056044 showed low potency (IC_50_, 10.5 μM) even though at full efficacy, while the other three compounds were inactive. Finally, these compounds were negative in an AmpC library profiling assay, indicating that they were not inhibitors of the β-lactamase reporter gene [26]. Taken together these results indicate that all four compounds showed selective inhibitory activity against the HIF-1 mediated signaling pathway, with marginal effect on the AP-1- and NFκB-mediated signaling pathways.

## Discussion

**Figure 6:**
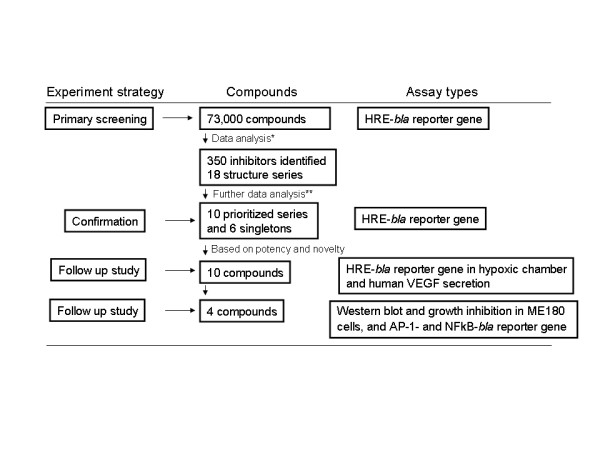
**A flowchart of identification of HIF-1 inhibitors**. * 350 inhibitory compounds were identified from primary screening, in which 18 structure series were generated by using Leadscope fingerprints. ** A set of criteria (Table S4, Additional file 5) were applied to exclude series containing potentially fluorescent compounds and series with low potencies or efficacies, resulting in 10 prioritized series and 6 singletons (Table S1, Additional file 1).

In the present study, we have engineered a ME180 cell line with a β-lactamase reporter under the control of a HRE that enables a reagent addition-only and microvolume (7 μL) screening assay for identifying small molecule inhibitors of the canonical HIF-1 signaling pathway. We have identified and confirmed compounds from several novel structural series that are inhibitors of the HIF-1 mediated pathway using a qHTS approach. These compounds showed good potency/efficacy, specificity against this pathway with no or low cytotoxicity, therefore might be good candidates for further testing in other cancer cell lines or animal models. The screening process and follow up studies were summarized as a flow chart in Figure 6.

Assays designed for the identification of modulators of the hypoxia pathway can be developed from individual pathway proteins or cell-based systems. The former biochemical assay allows identification of compounds which, for example, directly bind to the HIF-1 protein [27], but absent the cellular context which may influence *in vitro *cell activity or regulation. Therefore pathway assays can provide additional opportunities to modulate the target activity, although can require considerable follow-up studies to determine the specific site of action of the compound. However, screening a compound collection against a cell-based HIF-1 assay, such as the β-lactamase reporter gene assay, allows the identification of cell membrane permeable HIF-1 inhibitors that can be rapidly validated in secondary cell assays such as the hypoxia-induced HIF-1α accumulation and VEGF secretion assays used in this study.

The use of β-lactamase as a reporter with the cell-permeable substrate, CCF4, permits the measurement of the hypoxia response in live cells. The ratiometric readout by using this β-lactamase substrate and dual emissions (460 and 530 nm) minimizes the well-to-well and plate-to-plate variations caused by subtle differences in cell numbers that can result in the automated dispensing step. The green fluorescence signal (530 nm) is reflective of the cellular uptake of CCF4-AM substrate and can be used as an indication of cell viability. Therefore, it can be used to monitor compound cytotoxicity, independently from the response due to β-lactamase inhibition reflective of the pathway activity. For example, chetomin, a known HIF-1 inhibitor [28] with an IC_50 _value of 37.5 nM in this assay, was found to cause cytotoxicity at a concentration >200 nM under the induction of HIF-1 activity via CoCl_2 _[29]. The green fluorescence decreased as well as the blue fluorescence (460 nm) for this compound, indicating that chetomin inhibited CoCl_2 _induced HIF-1 activity and resulted in cytotoxicity at high concentrations.

As the cell-based HIF-1 assay, conducted in the presence of CoCl_2_, involves the activation of a β-lactamase reporter, it may identify a number of non-specific inhibitors that appear as positive either by competing with intracellular iron or inhibiting mRNA transcription, protein synthesis or β-lactamase activity. We have applied several follow-up assays including functional assays that measure hypoxia-induced HIF-1α accumulation and VEGF secretion, an HRE-*bla *assay conducted in a hypoxia chamber (≤1% oxygen), which serves as a confirmation assay of the CoCl_2_-induced HRE-*bla *assay, and an assay that detects compound cytotoxicity by measuring intracellular ATP content. In order to determine the specificity of the HIF-1 inhibitors identified from the primary screen and follow-up study, we also examined their activity in other signaling pathway assays run at the NCGC. The qHTS approach combined with several follow-up studies allowed us to efficiently identify the compounds capable of inhibiting the HIF-1 signaling pathway. Of the ten structural series and 6 singletons initially identified from qHTS, NCGC00043898, NCGC00044926, NCGC00049606 and NCGC00056044 had their activity confirmed in all follow-up studies, displaying potencies ranging from 0.5-3 μM. These compounds have not been reported previously as inhibitors of the HIF-1 signaling pathway and appeared to be selective toward this pathway as compared to the NFκB and AP-1 signaling pathways. In addition, we have demonstrated that these compounds blocked VEGF secretion, a downstream response to activated HIF-1.

Using the qHTS approach potency and efficacy of each compound can be obtained rapidly from the primary screening data, limiting the amount of follow-up compound acquisition and re-testing required. Confirmation rates are greatly improved in qHTS compared to conventional single concentration screening. For example, in our follow-up studies 36 of 40 compounds re-tested positive, a confirmation rate of 90%. We also confirmed thirteen of the thirteen compounds in the NCI diversity collection identified previously by two independent laboratories [23,30] (Table S3, Additional file 4), although the methods of hypoxia induction were different among all three labs. Of these compounds, NSC-259968 and NSC-259969 showed a potent inhibitory effect on the HIF-1 signaling pathway with IC_50_s of 11.3 and 10.9 nM. Two other compounds that were active are the DNA topoisomerase I inhibitors, NSC-606985 (camptothecin) and NSC-609699 (topotecan) with IC_50_s of 69 nM and 240 nM, respectively. Chau et al [23] excluded NSC-606985 and NSC-609699 as potential inhibitors of HIF-1 signaling due to their unacceptable cytotoxicity. We found that all four compounds not only displayed cytotoxicity as shown by a decrease in the green fluorescence signal (530 nm), but were also nonselective, as they blocked other pathways such as the AP-1 and NF-κ-B pathways as well as the HIF-1 signaling pathway (data not shown). The cytotoxicity of these compounds could not be detected in the HRE-luciferase reporter gene assay where they were identified as active because both cytotoxicity and inhibition of HIF-1 pathway would both result in a decrease in signal, thus the two modes are indistinguishable in this assay format.

## Conclusion

In summary, we have identified several novel small molecule inhibitors of the HIF-1 signaling pathway by applying qHTS to a HRE-*bla *assay and accessing the NIH Molecular Libraries Small Molecule Repository. Activities of selected compounds were confirmed in the follow-up assays. Among these compounds, NCGC00043898, NCGC00044926, NCGC00049606 and NCGC00056044 selectively inhibited both HIF-1 activity and VEGF protein expression. Three of these compounds showed inhibition of hypoxia-induced HIF-1α accumulation. Therefore, the screening approach outlined here has allowed us to identify high quality lead compounds that can serve as probes of the HIF-1 signaling pathway and as potential candidates for future drug development.

## Materials and methods

### Cell lines and culture conditions

CellSensor^® ^HRE-*bla *ME-180 cell line, generated in this study, stably expresses a β-lactamase reporter gene under the control of 5 copies of the HRE present in the VEGF gene promoter. CellSensor^® ^NFκB -*bla *and AP-1-*bla *ME-180 cell lines were obtained from Invitrogen (Invitrogen, Carlsbad, CA). CellSensor^® ^NFκB-*bla *ME-180 cells and CellSensor^® ^AP-1 (activator protein 1)-*bla *ME-180 cells contain a β-lactamase reporter gene under the control of the NFκB response element or the AP-1 binding element, respectively. Cells were cultured in DMEM medium supplemented with 10% dialyzed fetal bovine serum, 2 mM L-glutamine, 0.1 mM non-essential amino acids, 1 mM sodium pyruvate, 25 mM HEPES, 50 U/ml penicillin and 50 μg/ml streptomycin, and 5 μg/ml of blasticidin at 37°C under a humidified atmosphere and 5% CO_2_.

### HIF-1 RNAi assay and Western blot analysis

Cells were plated into a 96-well assay plate at 8000 cells/100 μL/well in growth medium and after overnight incubation at 37°C, 80 nM Stealth RNAi™ (Invitrogen) duplexes in Lipofactamine™ 2000 (Invitrogen) in OPTI-MEM was added. Cells were incubated for 48 hrs, the medium was replaced with OPTI-MEM plus 0.5% dialyzed FBS, and the indicated amount of CoCl_2 _or deferoxamine (DFO) added. After 17 hrs incubation at 37°C, β-lactamase activity was measured.

For Western blot analysis, cells were plated in 6-well plate, mock transfected or transfected with indicated Stealth RNAi™ oligos as described above for 55 hrs, and then were left untreated or treated with 200 uM CoCl_2 _for 5 hrs. Cells were harvested and incubated with M-PER cell lysis buffer (Thermo Scientific) supplemented with protease inhibitor cocktail (Sigma, St. Louis, MO) and DNAase I (Invitrogen) for 15 min on ice. Samples for SDS-PAGE were prepared by addition of 5× SDS loading buffer and DTT (1 mM) to the cell lysates. Samples were boiled at 90°C for 3 min and then loaded onto a 4-20% Tris-Glycine gel. Resolved proteins were transferred to nitrocellulose membrane using the iBlot semi-dry transfer unit (Invitrogen). The western blotting was then performed using primary anti-HIF-1α antibody (Cell Signaling Technologies, Danvers, MA) and alkaline-phosphatase conjugated secondary goat anti-rabbit antibody (Invitrogen). Reactive bands were detected using a chromogenic substrate reagent (BCIP/NBT) (Invitrogen).

### HRE β-lactamase reporter assay

HRE-*bla *ME-180 cells were dissociated with 0.05% trypsin/EDTA, washed in assay medium (OPTI-MEM with 0.5% fetal bovine serum, 0.1 mM nonessential amino acids, 1 mM sodium pyruvate, 10 mM HEPES pH 7.3, 100 U/ml penicillin, and 100 μg/ml streptomycin), and suspended in assay medium. As described in Table 3, cells were dispensed at 2500 cells/5 μL/well in 1536-well black wall/clear bottom plates (Greiner Bio-One North America, Monroe, NC) using a Flying Reagent Dispenser [31] (FRD, Aurora Discovery, Carlsbad, CA). Twenty-three nL of compound was transferred to the assay plate by a pin tool [32] (Kalypsys, San Diego, CA) resulting in a 261-fold dilution. One μL of medium with or without agonist or agonist plus inhibitor was dispensed by a FRD in the following format. Column 1, NSC 607097 [23], a known inhibitor of HIF-1 pathway, concentration-response titration from 0.6 nM to 2 μM, column 2, 120 μM CoCl_2 _(EC_100_), column 3, DMSO only, and columns 1, 4 to 48, 60 μM CoCl_2_. The plates were incubated 17 hours at 37°C. One μL of LiveBLAzer™ B/G FRET substrate (Invitrogen) detection mixture was added, the plates were incubated at room temperature for 2.5 hrs, and fluorescence intensity (405 nm excitation, and 460 and 530 nm emissions) was measured by an Envision plate reader (Perkin Elmer, Shelton, CT). Data was expressed as the ratio of 460/530 nm emissions.

**Table 3:** qHTS protocol

Step	Parameter	Value	Description
1	Plate cells	5 μL	2500 HRE-*bla *Cells
2	Incubation time	4-6 hr	Cells adhere and acclimate
3	Library Compound Control Compound	23 nL 23 nL	38 μM to 0.5 nM titration series 0.1 nM to 2 μM titrations (NSC 607097)
4	Reagent	1 μL	Buffer +/- agonist
5	Incubation time	17 hrs	Induce HRE reporter gene
6	Reagent	1 μL	β-Lactamase detection mixture
7	Incubation	2.5 hrs	Cells load and cleave dye
8	Assay Readout	Ex = 405/8 nm	Envision

Notes

1 Black clear bottom 1536-well plates, single tip dispense of 2500 cells/well into all wells

2 37°C, 5% CO_2 _incubator

3 Pintool transfer of library to columns 5-48, and controls to columns 1-4.

4 Single tip dispense for each reagent, Column 1 and 4-48, agonist-60 μM CoCl2. Column 2, agonist-120 μM CoCl_2_. Column 3, buffer only.

5 37°C, 5% CO_2 _incubator

6 Single tip dispense of 0.6 μM CCF4-AM, 1 mg/ml Pluronic F127 surfactant, 3.5% PEG 400, 2.6% TR40, 2 mM probenecid, 0.1% DMSO

8 460/25 nm and 530/20 nm emission filters

In the confirmation study, the selected compounds were first tested in HRE-*bla *assay induced by 60 μM CoCl_2_. The assay protocol is same as above described except the dose titrations were within one 1536-well plate and compounds were tested at 24 concentrations in duplicate. In the compound follow up, the activity of compounds was further confirmed in HRE-*bla *assay in the presence of ≤1% oxygen in a hypoxia chamber (Billups-Rothenberg, Inc, CA). The process of compound selection will be described in the following section.

### qHTS and SAR analysis

Approximately 73,000 compounds were screened in this qHTS [18,19,33]. Eighty-two percent of these compounds come from the Molecular Libraries Screening Centers Network (MLSCN) library collection, 15% from targeted libraries, 1% from natural products and 2% from known bioactives. Compound plates were prepared as inter-plate titrations of at least seven dilutions with the four left-most columns left empty in each plate [34]. Pin tool transfer of compounds to assay plates resulted in a 261-fold dilution. The final compound concentration in the 6 μl assay volume ranged from 0.0024 to 38 μM. Primary data analysis was performed as previously described [18]. Briefly, raw plate reads for each titration point were first normalized relative to CoCl_2 _control (60 μM, 100%) and DMSO only wells (basal, 0%), and then corrected by applying a pattern correction algorithm using compound-free control plates (DMSO plates) between each library of the compound plate stack. Concentration-response titration points for each compound were fitted to the Hill equation yielding concentrations of half-maximal activity (IC_50_) and maximal response (efficacy) values. Compounds from qHTS were designated as Class 1-4 according to the type of concentration-response curve observed [18]. Curve classes are heuristic measures of data confidence, classifying concentration responses on the basis of efficacy, number of data points showing above background activity, and the quality of fit. Briefly, compounds that did not show any concentration response or had no significant activity point were classified as Class 4; compounds only displayed significant activity at the highest tested concentration were classified as Class 3; and compounds with other types of concentration-response curves were classified as either Class 1 - compounds with complete response curves, or Class 2 - compounds with incomplete response curves. Compounds with Class 1 or 2 curves were further divided into subclasses based on efficacy and quality of fit (*r*^2^). Compounds with good quality curves and high efficacies (>80%) were designated as subclass 1.1 or 2.1, and low but significant efficacies (30-80%) as subclass 1.2 or 2.2. Compounds with Class 1.1, 1.2 or 2.1 curves were generally selected for follow up analyses as they represented high confidence data. Compounds with Class 4 curves in the 530 nm reading, Class 1-3 in the 460 nm reading, and Class 1.1, 1.2 or 2.1 in the ratio determination were defined as active inhibitors and selected for structure-activity relationships (SAR) analysis. To identify active scaffolds, selected compounds were clustered using Leadscope^® ^fingerprints, yielding 72 clusters. Maximal common substructures (MCS) were extracted from each cluster containing at least 4 active compounds, which were then used to search the entire screening collection to find all analogs, including inactives, yielding 18 series (compounds sharing a common scaffold formed a series). A set of criteria (Table S4, Additional file 5) were applied to exclude series containing potentially fluorescent compounds and series with low potencies or efficacies, resulting in 10 prioritized series and 6 singletons.

### Measurement of VEGF secretion

HRE-*bla *cells were plated in growth medium at 1 × 10^5 ^cells/well in a 24-well plate. After incubation for 3-5 hrs at 37°C, the cell culture medium was removed and OPTI-MEM medium with 1% dialyzed FBS was added into the wells. The cells were then treated with compound for 5 min, followed by addition of 15 μM of iodochorohydrozyquinoline or 60 μM CoCl_2_. After 20 hr treatment, the culture media were removed and analyzed for VEGF expression using human VEGF immunoassay kit (R&D Systems, Minneapolis, MN). Briefly, 200 uL of sample or known standard (0-2000 pg/ml) was added to wells of a microplate which was pre-coated with a monoclonal antibody specific VEGF and incubated at room temperature for 2 hrs. After washing away any unbound substances, an anti-VEGF antibody conjugated to horseradish peroxidase was added and the plate incubated for 2 hrs at room temperature. Following three washes, a substrate solution was added and incubated for 20 min, followed by the addition of a stop solution. The optical density of each well was determined using an EnVision plate reader at 450 nm with 570 nm as a reference filter.

### NFκB and AP-1 β-lactamase reporter assays

NFκB-*bla *or AP-1-*bla *ME-180 cells were dissociated with 0.05% trypsin/EDTA, resuspended in assay medium, and dispensed at 2000-2500 cells/5 μL/well in 1536-well black wall/clear bottom plates (Greiner Bio-One North America) using a FRD. Twenty-three nL of compound was transferred to the assay plate by a pin tool (Kalypsys) resulting in a 261-fold dilution. For NFκB assay, 1 μL of medium with or without 1 ng/ml TNF-α was dispensed by a FRD, and for AP-1 assay 1 μL of medium with or without 1 ng/ml EGF was dispensed by a FRD. After the plates were incubated 5 hrs at 37°C, one μL of LiveBLAzer™ B/G FRET substrate (Invitrogen, CA) detection mixture was added. The plates incubated at room temperature for 2 hrs, and fluorescence intensity (405 nm excitation, 460 nm and 530 nm emissions) was measured by an Envision plate reader. Data was expressed as the ratio of 460/530 nm emissions.

### Cell viability assay

Cell viability was measured using a luciferase-coupled ATP quantitation assay (CellTiter-Glo^®^, Promega, Madison, WI). Cells were dispensed at 2500 cells/well in 1536-well white/solid bottom assay plates (Greiner Bio-One North America) using a FRD. The cells were incubated at 37°C for 4-6 hrs to allow for cell attachment, followed by addition of compounds via pin tool. After compound addition, plates were incubated for 17-72 hrs at 37°C. At the end of the incubation period, 5 μL of CellTiter-Glo^® ^reagent was added, plates were incubated at room temperature for 30 min, and luminescence intensity determined using a ViewLux plate reader (PerkinElmer, Shelton, CT).

## Competing interests

The authors declare that they have no competing interests.

## Authors' contributions

MX, JI, KB, CPA and JP designed research; MX, KB, MHC, SS and SCM performed experiments; RH, MX, KB and JI analyzed data; MX, KB, RH, JP, CPA and JI wrote the paper; All authors read the approved the final manuscript.

## Supplementary Material

Additional file 1 **Table S1. Compounds selected for confirmation study**. Additional table. Click here for file

Additional file 2 **Figure S1. Inhibitory effect of compounds on CoCl_2_-induced HIF-1α accumulation**. Additional figure. Click here for file

Additional file 3 **Table S2. Potency (μM, IC_50_) and efficacy (% of inhibition) of compounds at different treatment time in cytotoxicity assay in absence or presence of CoCl_2_**. Additional table. Click here for file

Additional file 4 **Table S3. Comparison of compounds identified in this screening with those identified by Chau et al. **[23]** and Rapisarda et al. **[30]. Additional table. Click here for file

Additional file 5 **Table S4. Series prioritization criteria**. Additional table. Click here for file

## References

[B1] Mole DR Ratcliffe PJ Cellular oxygen sensing in health and disease Pediatr Nephrol 2008 23 681 94 10.1007/s00467-007-0632-x 17955264

[B2] Wang GL Semenza GL Purification and characterization of hypoxia-inducible factor 1 J Biol Chem 1995 270 1230 7 10.1074/jbc.270.3.1230 7836384

[B3] Huang LE Gu J Schau M Bunn HF Regulation of hypoxia-inducible factor 1alpha is mediated by an O2-dependent degradation domain via the ubiquitin-proteasome pathway Proc Natl Acad Sci USA 1998 95 7987 92 10.1073/pnas.95.14.7987 9653127 PMC20916

[B4] Salceda S Caro J Hypoxia-inducible factor 1alpha (HIF-1alpha) protein is rapidly degraded by the ubiquitin-proteasome system under normoxic conditions. Its stabilization by hypoxia depends on redox-induced changes J Biol Chem 1997 272 22642 7 10.1074/jbc.272.36.22642 9278421

[B5] Ivan M Kondo K Yang H Kim W Valiando J Ohh M Salic A Asara JM Lane WS Kaelin WG Jr HIFalpha targeted for VHL-mediated destruction by proline hydroxylation: implications for O2 sensing Science 2001 292 464 8 10.1126/science.1059817 11292862

[B6] Jaakkola P Mole DR Tian YM Wilson MI Gielbert J Gaskell SJ Kriegsheim A Hebestreit HF Mukherji M Schofield CJ Targeting of HIF-alpha to the von Hippel-Lindau ubiquitylation complex by O2-regulated prolyl hydroxylation Science 2001 292 468 72 10.1126/science.1059796 11292861

[B7] Lando D Peet DJ Gorman JJ Whelan DA Whitelaw ML Bruick RK Asparagine hydroxylation of HIF transactivation domain: a hypoxic switch Science 2002 295 858 861 10.1126/science.1068592 11823643

[B8] Forsythe JA Jiang BH Iyer NV Agani F Leung SW Koos RD Semenza GL Activation of vascular endothelial growth factor gene transcription by hypoxia-inducible factor 1 Mol Cell Biol 1996 16 4604 13 8756616 10.1128/mcb.16.9.4604 PMC231459

[B9] Guillemin K Krasnow MA The hypoxic response: huffing and HIFing Cell 1997 89 9 12 10.1016/S0092-8674(00)80176-2 9094708

[B10] Chen C Pore N Behrooz A Ismail-Beigi F Maity A Regulation of glut1 mRNA by hypoxia-inducible factor-1. Interaction between H-ras and hypoxia J Biol Chem 2001 276 9519 25 10.1074/jbc.M010144200 11120745

[B11] Iyer NV Kotch LE Agani F Leung SW Laughner E Wenger RH Gassmann M Gearhart JD Lawler AM Yu AY Semenza GL Cellular and developmental control of O2 homeostasis by hypoxia-inducible factor 1 alpha Genes Dev 1998 12 149 62 10.1101/gad.12.2.149 9436976 PMC316445

[B12] Redell MS Tweardy DJ Targeting transcription factors for cancer therapy Curr Pharm Des 2005 11 2873 87 10.2174/1381612054546699 16101443

[B13] Ryan HE Lo J Johnson RS HIF-1 alpha is required for solid tumor formation and embryonic vascularization Embo J 1998 17 3005 15 10.1093/emboj/17.11.3005 9606183 PMC1170640

[B14] Tang N Wang L Esko J Giordano FJ Huang Y Gerber HP Ferrara N Johnson RS Loss of HIF-1alpha in endothelial cells disrupts a hypoxia-driven VEGF autocrine loop necessary for tumorigenesis Cancer Cell 2004 6 485 95 10.1016/j.ccr.2004.09.026 15542432

[B15] Garber K New drugs target hypoxia response in tumors J Natl Cancer Inst 2005 97 1112 4 16077066 10.1093/jnci/dji261

[B16] Nagle DG Zhou YD Natural product-derived small molecule activators of hypoxia-inducible factor-1 (HIF-1) Curr Pharm Des 2006 12 2673 88 10.2174/138161206777698783 16842166 PMC2907550

[B17] Semenza GL Targeting HIF-1 for cancer therapy Nat Rev Cancer 2003 3 721 32 10.1038/nrc1187 13130303

[B18] Inglese J Auld DS Jadhav A Johnson RL Simeonov A Yasgar A Zheng W Austin CP Quantitative high-throughput screening: a titration-based approach that efficiently identifies biological activities in large chemical libraries Proc Natl Acad Sci USA 2006 103 11473 8 10.1073/pnas.0604348103 16864780 PMC1518803

[B19] Xia M Huang R Guo V Southall N Cho MH Inglese J Austin CP Nirenberg M Identification of compounds that potentiate CREB signaling as possible enhancers of long-term memory Proc Natl Acad Sci USA 2009 106 2412 7 10.1073/pnas.0813020106 19196967 PMC2638736

[B20] Triantafyllou A Liakos P Tsakalof A Georgatsou E Simos G Bonanou S Cobalt induces hypoxia-inducible factor-1alpha (HIF-1alpha) in HeLa cells by an iron-independent, but ROS-, PI-3K- and MAPK-dependent mechanism Free Radic Res 2006 40 847 56 10.1080/10715760600730810 17015263

[B21] Maxwell P Salnikow K HIF-1: an oxygen and metal responsive transcription factor Cancer Biol Ther 2004 3 29 35 14726713 10.4161/cbt.3.1.547

[B22] Vukovic V Haugland HK Nicklee T Morrison AJ Hedley DW Hypoxia-inducible factor-1alpha is an intrinsic marker for hypoxia in cervical cancer xenografts Cancer Res 2001 61 7394 8 11606368

[B23] Chau NM Rogers P Aherne W Carroll V Collins I McDonald E Workman P Ashcroft M Identification of novel small molecule inhibitors of hypoxia-inducible factor-1 that differentially block hypoxia-inducible factor-1 activity and hypoxia-inducible factor-1alpha induction in response to hypoxic stress and growth factors Cancer Res 2005 65 4918 28 10.1158/0008-5472.CAN-04-4453 15930314

[B24] Chiarugi V Magnelli L Chiarugi A Gallo O Hypoxia induces pivotal tumor angiogenesis control factors including p53, vascular endothelial growth factor and the NFkappaB-dependent inducible nitric oxide synthase and cyclooxygenase-2 J Cancer Res Clin Oncol 1999 125 525 8 10.1007/s004320050312 10480347 PMC12172383

[B25] Xia M Huang R Sun Y Semenza GL Aldred SF Witt K Inglese J Tice RR Austin CP Identification of chemical compounds that induce HIF-1α activity Toxicological Sciences 2009 112 153 163 10.1093/toxsci/kfp123 19502547 PMC2910898

[B26] Babaoglu K Simeonov A Irwin JJ Nelson ME Feng B Thomas CJ Cancian L Costi PM Maltby DA Jadhav A Inglese J Austin CP Shoichet BK Comprehensive mechanistic analysis of hits from high-throughput and docking screens against β-lactamase J Med Chem 2008 51 2502 2511 10.1021/jm701500e 18333608 PMC2655312

[B27] Kong D Park EJ Stephen AG Calvani M Cardellina JH Monks A Fisher RJ Shoemaker RH Melillo G Echinomycin, a small-molecule inhibitor of hypoxia-inducible factor-1 DNA-binding activity Cancer Res 2005 65 9047 55 10.1158/0008-5472.CAN-05-1235 16204079

[B28] Kung AL Zabludoff SD France DS Freedman SJ Tanner EA Vieira A Cornell-Kennon S Lee J Wang B Wang J Small molecule blockade of transcriptional coactivation of the hypoxia-inducible factor pathway Cancer Cell 2004 6 33 43 10.1016/j.ccr.2004.06.009 15261140

[B29] Bi K Xia M Allred J Inglese J Printen J A high throughput cell-based assay for interrogating hypoxia-induced signaling pathway Drug Discovery Technology and Development: 8-11. August 2005; Boston

[B30] Rapisarda A Uranchimeg B Scudiero DA Selby M Sausville EA Shoemaker RH Melillo G Identification of small molecule inhibitors of hypoxia-inducible factor 1 transcriptional activation pathway Cancer Res 2002 62 4316 24 12154035

[B31] Niles WD Coassin PJ Piezo- and solenoid valve-based liquid dispensing for miniaturized assays Assay Drug Dev Techno 2005 3 189 202 10.1089/adt.2005.3.189 15871693

[B32] Cleveland PH Koutz PJ Nanoliter dispensing for uHTS using pin tools Assay Drug Dev Technol 2005 3 213 25 10.1089/adt.2005.3.213 15871695

[B33] Michael S Auld D Klumpp C Jadhav A Zheng W Thorne N Austin CP Inglese J Simeonov A A robotic platform for quantitative high-throughput screening Assay Drug Dev Technol 2008 6 637 57 10.1089/adt.2008.150 19035846 PMC2651822

[B34] Yasgar A Shinn P Jadhav A Auld D Michael S Zheng W Austin CP Inglese J Simeonov A Compound Management for Quantitative High-Throughput Screening JALA Charlottesv Va 2008 13 79 89 18496600 10.1016/j.jala.2007.12.004 PMC2390859

