# Supplementary material for: Identification of small molecule compounds that inhibit the HIF-1 signaling pathway
Source: Mol Cancer. 2009 Dec 9;8:117. doi: 10.1186/1476-4598-8-117 (PMC2797767; doi:10.1186/1476-4598-8-117)
Supplement: Additional file 1 — Table S1. Compounds selected for confirmation study. Additional table. [file 1476-4598-8-117-S1.DOC]

Additional files

Table S1. Compounds selected for confirmation study.

| **#** | **Sample ID** | **Structure** | **Series ID** | **Primary Screen IC50 (µM)** | **Confirmation Screen IC50 (µM)** | **PubChem SID** |
| --- | --- | --- | --- | --- | --- | --- |
| 1 | NCGC00055619-01 |  | 8 | 1.3 | 10.0 | 4255655 |
| 2 | NCGC00085878-02 |  | 8 | 1.3 | 11.2 | 7965457 |
| 3 | NCGC00013003-01 |  | 8 | 2.5 | 12.6 | 4252450 |
| 4 | NCGC00078957-01 |  | 8 | 0.8 | Inactive | 7973071 |
| 5 | NCGC00013538-01 |  | 11 | 0.8 | 2.0 | 4252985 |
| 6 | NCGC00016391-01 |  | 11 | 2.0 | 2.0 | 11112285 |
| 7 | NCGC00044187-01 |  | 21 | 1.0 | 5.0 | 4246877 |
| 8 | NCGC00025330-01 |  | 21 | 2.5 | 7.9 | 11114251 |
| 9 | NCGC00060755-01 |  | 21 | 1.3 | Inactive | 4260302 |
| 10 | NCGC00078922-01 |  | 23 | 1.0 | 6.3 | 7975186 |
| 11 | NCGC00026810-01 |  | 23 | 2.5 | 10.0 | 852691 |
| 12 | NCGC00033914-01 |  | 23 | 4.0 | 10.0 | 853532 |
| 13 | NCGC00041538-01 |  | 27 | 1.0 | 0.9 | 3712283 |
| 14 | NCGC00049423-01 |  | 27 | 2.0 | 1.1 | 4244475 |
| 15 | NCGC00043887-01 |  | 27 | 0.1 | 1.6 | 4247289 |
| 16 | NCGC00043836-01 |  | 27 | 0.1 | 2.5 | 4249058 |
| 17 | NCGC00044926-01 |  | 31 | 1.3 | 0.5 | 4243018 |
| 18 | NCGC00043631-01 |  | 31 | 2.0 | 0.5 | 4240727 |
| 19 | NCGC00038673-01 |  | 31 | Inactive | Inactive | 3711970 |
| 20 | NCGC00044763-01 |  | 39 | 2.5 | 2.5 | 4250431 |
| 21 | NCGC00043897-01 |  | 39 | 5.0 | 2.5 | 4245724 |
| 22 | NCGC00043898-01 |  | 39 | 2.0 | 2.8 | 4246346 |
| 23 | NCGC00049606-01 |  | 39 | 12.6 | 3.2 | 4252099 |
| 24 | NCGC00040130-01 |  | 39 | 0.8 | 4.0 | 3712742 |
| 25 | NCGC00052277-01 |  | 42 | 0.8 | 12.6 | 4241708 |
| 26 | NCGC00045026-01 |  | 42 | 10.0 | 17.8 | 4241848 |
| 27 | NCGC00045025-02 |  | 42 | Inactive | Inactive | 4242820 |
| 28 | NCGC00026542-01 |  | 48 | 4.0 | 4.0 | 849732 |
| 29 | NCGC00033021-01 |  | 48 | 2.0 | 5.0 | 847599 |
| 30 | NCGC00028456-01 |  | 48 | 4.0 | 5.0 | 848285 |
| 31 | NCGC00076418-01 |  | 48 | 5.0 | 7.1 | 7972842 |
| 32 | NCGC00024283-01 |  | 48 | 2.0 | 17.8 | 859166 |
| 33 | NCGC00025172-01 |  | 48 | Inactive | Inactive | 11114093 |
| 34 | NCGC00056044-01 |  | 71 | 0.4 | 3.2 | 4265248 |
| 35 | NCGC00033933-01 |  | Singleton | 0.0 | 0.0 | 846218 |
| 36 | NCGC00041984-01 |  | Singleton | 1.0 | 3.2 | 3711096 |
| 37 | NCGC00039242-01 |  | Singleton | 0.6 | 5.0 | 3711663 |
| 38 | NCGC00053249-01 |  | Singleton | 1.0 | 5.6 | 4261193 |
| 39 | NCGC00061830-01 |  | Singleton | 0.8 | 12.6 | 4261439 |
| 40 | NCGC00069309-01 |  | Singleton | 0.8 | Inactive | 860897 |
