# Supplementary material for: Identification of small molecule compounds that inhibit the HIF-1 signaling pathway
Source: Mol Cancer. 2009 Dec 9;8:117. doi: 10.1186/1476-4598-8-117 (PMC2797767; doi:10.1186/1476-4598-8-117)
Supplement: Additional file 2 — Figure S1. Inhibitory effect of compounds on CoCl2-induced HIF-1α accumulation. Additional figure. [file 1476-4598-8-117-S2.DOC]

Additional files

Figure S1. Inhibitory effect of compounds on CoCl_2_ induced HIF-1α accumulation.

HRE-*bla* ME-180 cells were treated DMSO only (lane 1), 80 µM CoCl_2_ (lane 2), 10 µM NCGC00043989 (lane 3), 20 µM NCGC00043989 (lane 4), 10 µM NCGC00044926 (lane 5), 20 µM NCGC00044926 (lane 6), 10 µM NCGC00049606 (lane 7), 20 µM NCGC00049606 (lane 8), 10 µM NCGC00056044 (lane 9) and 20 µM NCGC00056044 (lane 10) for 24hr. The compound treatment was in the presence of 80 µM CoCl_2_ which is optimal concentration for the Western blot. Cells were harvested and lysed in a lysis buffer (5mM Tris-HCl, pH 8, 20mM EDTA and 0.5% Triton X-100) with protease inhibitor (Roche) for 30 min on ice. Supernatants were collected after centrifugation in a bench top centrifuge (14,000 rpm) at 4ºC and subjected to SDS-PAGE analysis on an 8% Tris-Glycine gel. The proteins were then transferred to a membrane and probed with antibodies against HIF-1 (Santa Cruz) and β-Actin (Sigma) as a loading control.
