# Supplementary material for: Identification of small molecule compounds that inhibit the HIF-1 signaling pathway
Source: Mol Cancer. 2009 Dec 9;8:117. doi: 10.1186/1476-4598-8-117 (PMC2797767; doi:10.1186/1476-4598-8-117)
Supplement: Additional file 3 — Table S2. Potency (μM, IC50) and efficacy (% of inhibition) of compounds at different treatment time in cytotoxicity assay in absence or presence of CoCl2. Additional table. [file 1476-4598-8-117-S3.DOC]

Additional files

Table S2. Potency (µM, IC_50_) and efficacy (% of inhibition) of compounds at different treatment time in cytotoxicity assay in absence or presence of CoCl_2_
