# Supplementary material for: Identification of small molecule compounds that inhibit the HIF-1 signaling pathway
Source: Mol Cancer. 2009 Dec 9;8:117. doi: 10.1186/1476-4598-8-117 (PMC2797767; doi:10.1186/1476-4598-8-117)
Supplement: Additional file 4 — Table S3. Comparison of compounds identified in this screening with those identified by Chau et al. [23] and Rapisarda et al. [30]. Additional table. [file 1476-4598-8-117-S4.DOC]

Additional files

Table S3. Comparison of compounds identified in this screening with those identified by Chau et al. [23] and Rapisarda et al. [30].

|  | Chau et al. [23] | Rapisarda et al. [28] | IC50 (nM) |
| --- | --- | --- | --- |
| NSC-134754 | + | - | - (15% inhibition) |
| NSC-607097 | + | + | 37 |
| NSC-643735 | + | + | 13 |
| NSC-675865 | + | + | 11.8 |
| NSC-259968 | + | + | 11.3 |
| NSC-259969 | + | + | 10.9 |
| NSC-25485 | + | + | 40.9 |
| NSC-131547 | + | + | 250 |
| NSC-609699 | - | + | 214 |
| NSC-606985 | - | + | 65 |
| NSC-639174 | - | + | 779 |
| NSC-359449 | - | + | 5100 |
| NSC-254681 | - | + | 31800 |

Note: +, the compounds were identified by either Chau et al or Rapisard et al;

-, compound had not been identified by these two labs.
