# Supplementary material for: Identification of small molecule compounds that inhibit the HIF-1 signaling pathway
Source: Mol Cancer. 2009 Dec 9;8:117. doi: 10.1186/1476-4598-8-117 (PMC2797767; doi:10.1186/1476-4598-8-117)
Supplement: Additional file 5 — Table S4. Series prioritization criteria. Additional table. [file 1476-4598-8-117-S5.DOC]

Additional files

Table S4. Series prioritization criteria.

| Attribute | Reference Assay Activity | Requirement for Selection |
| --- | --- | --- |
| Fluorescence | Activation in green channel (530 nm); spectroscopic profiling | Negative |
| Cytotoxicity | Inhibition in green channel (530 nm) | Negative |
| Selectivity | Activity in other BLA assays and other assays screened in NCGC | Less than 3 assays |
| Potency/efficacy | Ratio channel | Most potent compound in series: IC50<1 µM; efficacy>50% |
| Enrichment of active compounds | Ratio channel | Not significantly deficient of active compounds |
